# Development of a low-pressure microtargeting biolistic device for transfection of retinal explants

**Published:** 2011-11-16

**Authors:** Melissa G. Christianson, Donald C. Lo

**Affiliations:** Center for Drug Discovery and Department of Neurobiology, Duke University Medical Center, Durham, NC

## Abstract

Biolistic transfection offers a key experimental method for molecular perturbation of bona fide, postmitotic neurons within their native local environment in explanted tissues. However, current, commercially available biolistic devices unavoidably deliver traumatic injury to surface layers of explanted tissues because of helium co-emission with DNA-coated gold particles during the shooting process. This makes such methods unsuitable for use with the delicate tissue layers of the mammalian retina. Here, we report the development of a novel and inexpensive microtargeting biolistic device that avoids the trauma associated with conventional entrainment biolistic methods, permitting rapid and efficient transfection of retinal ganglion cells in the adult mammalian retina without significant damage to their local microenvironment. By using low helium inflow pressures and vacuum diversion to eliminate helium emission during the transfection process, we found that the current method allowed efficient transfection as well as morphological and functional preservation of retinal ganglion cells and their local glial microenvironment in transfected retinal explants from adult rats. The use of an ethanol-gold suspension further supported rapid and extended shooting sequences and reduced shot-to-shot variation during transfection compared to existing tubing-based devices. This new biolistic device should be useful not only in the retina, but also in other tissue explant settings in which preservation of local cellular and tissue integrity is a priority.

## Introduction

The mammalian retina offers a uniquely structured tissue region of the central nervous system in which a diverse range of neuronal and glial cells types is easily accessible for experimental studies [1,2]. Retinal ganglion cells (RGCs) in particular have served as a useful model of a central nervous system projection neuron: In vivo, the axons of the RGCs bundle to form the optic nerve, which exits at the rear of the ocular globe as it extends toward its targets in the lateral geniculate nucleus of the thalamus. RGCs elaborate their extensive dendritic arbors primarily within a single layer of the retina, and are supported by a web of astrocytes overlying the nerve fiber layer, as well as by Müller glial cells spanning the full thickness of the retina. This distinctive neuronal-glial architecture provides a spatially organized system in which neuronal and axonal function can be studied in the presence of the critical supporting glial cell matrix. Recent work has increasingly exploited the advantages of this structured system for the study of neuronal and glial actions in retinal explants ex vivo [3-11].

Particle-mediated transfection, or biolistics, has been used extensively to transfect postmitotic neurons in neural tissue explants [12-14] and to label RGCs in explanted retinas with fluorescent or genetic markers [15-17]. However, conventional biolistic transfection methods are unavoidably accompanied by traumatic injury to surface tissue layers, caused by the high-pressure helium transients that are used to propel the DNA-coated gold particles (so-called “entrainment” devices [18]). Such damage is particularly troubling in biolistic transfection of retinal explants, as RGC axons and their astroglial support matrix reside in the most superficial layers of the retina. Physical damage to these layers thus compromises the three-dimensional environment of explanted RGCs and complicates the interpretation of experimental results. Yet, biolistics remains perhaps the only method that can be used to transfect RGCs in living retinal explants with any degree of efficiency. Other transfection methods, such as lipid-mediated transfection or the use of viral vectors, suffer from lack of spatial resolution and inconsistent transfection efficiencies in tissue explants. Electroporation protocols developed for use in the retina primarily target photoreceptors and bipolar cells and have seen only modest success in transfecting the RGC layer [19,20].

In this context, we have developed a novel and inexpensive microtargeting biolistic device that avoids the trauma associated with conventional entrainment biolistic methods, permitting rapid and efficient transfection of RGCs in the adult mammalian retina without damaging their local microenvironment.

## Methods

### Biolistic transfection with a modified capillary gun

**Figure 1:**
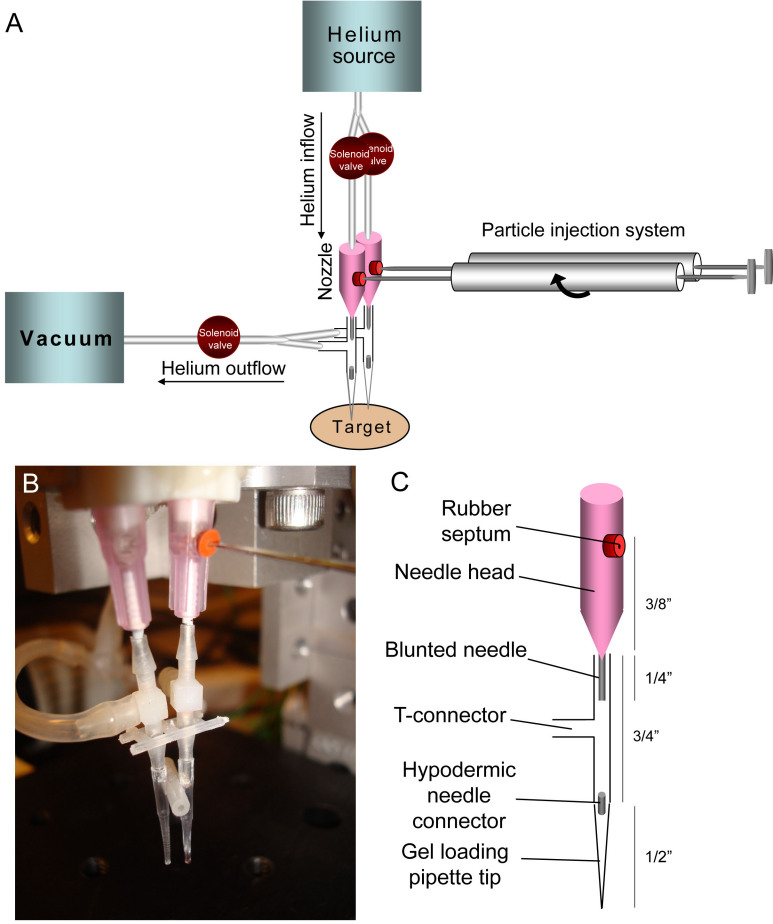
Modified capillary gun apparatus. **A**: The modified capillary gun was constructed according to the schematic shown above. **B**, **C**: The gun’s nozzle is made up of a blunted Luer lock disposable needle (18 gauge), plastic T-connector, and gel loading pipette tip, assembled as shown in the diagram. As described in more detail in the text, low-pressure helium flow is used to accelerate aliquots of DNA-coated gold microparticles suspended in ethanol that are injected into the head of the needle. As the microparticles emerge from the tip of the blunted needle, a vacuum flow is used to divert the bulk flow of helium through the side arm of the T-connector. As the flow rate of the vacuum just exceeds that of the helium inflow, the dense gold microparticles continue along their initial trajectory through the T- and hypodermic needle connectors to the gel loading pipette tip for emission. During this process, the ethanol is removed and/or evaporates such that the microparticles are dried by the time they reach the target retinal tissue after a brief flight through air.

We have modified the previously described a capillary gun [21] for rapid and efficient use in the explanted retina. The modified gun comprises a helium inflow/outflow system, particle injection system, and nozzle assembled onto an adjustable-height stage (Figure 1). During transfection, retinas explanted into 12-well plates are centered on the stage below the gun’s nozzle. The height of the stage can be adjusted to control the depth of particle penetration and thus the layer of cells to be transfected. Our current assembly is optimized to target the RGC layer in retinal explants from adult rats, using a nozzle-to-explant distance of approximately 0.6 cm.

Helium gas controlled by an inflow-outflow system is used to propel DNA-coated gold particles toward the explanted retina. Helium is injected into the gene gun assembly via flexible tubing attached to a compressed helium source. Depression of a trigger connected to a time-delay relay opens a solenoid valve and allows a 50 ms pulse of helium to flow into the gun apparatus. Notably, this pulse of helium enters the gun at pressures as low as 15 psi—the outflow pressure of the helium jet exiting from the gun is thus considerably reduced in comparison to traditional biolistic devices, which routinely employ helium inflow pressures of greater than 100 psi and as high as 2,000 psi [12,13,22]. This modest outflow pressure is further reduced by the use of active vacuum suctioning at the nozzle during the transfection process. Depression of the trigger simultaneously opens a second solenoid valve attached to a vacuum source via flexible tubing. This vacuum creates negative pressure at the gun’s nozzle to divert the flow of helium, but does not affect the continued inertial movement of the highly dense gold particles.

Like the previously developed capillary biolistic gene gun, the present device also uses an ethanol-gold particle suspension that is injected directly into the nozzle of the gun. Injections are made using a 100 μl Hamilton syringe (Hamilton Company, Reno, NV), which is continuously rotated using a custom-built K’Nex apparatus (Rodon Group, Hatfield, PA) to ensure even particle distribution and suspension before loading. A micromanipulator attached to the loading apparatus is used to depress the syringe plunger to deliver approximately 0.5 μl of the ethanol-gold particle suspension into the gun for each shot. As this small volume of gold suspension is propelled through the headpiece and nozzle by the jet of helium, the ethanol carrier evaporates such that gold particles exiting from the gun’s nozzle are dry.

The gun’s nozzle is made up of a Luer Lock–attached disposable needle (18 gauge; #305195; Becton, Dickinson and Company, Franklin Lakes, NJ) with the sharp tip removed (Figure 1B). A hole drilled into the head of the needle and plugged with a rubber septum (#WS-2; Warner Instruments, Hamden, CT) connects the nozzle to the Hamilton syringe of the particle-injection system described above. The remainder of the nozzle comprises a small T-connector (#T210–6; Small Parts Inc., Miami Lakes, FL) and a gel loading pipette tip (#BTGel30; Neptune Scientific, San Diego, CA). These pieces are connected to the blunted needle such that gas flows in a straight line from the needle’s headpiece straight through the shaft/T-connector/tip (Figure 1C). We have found that the relative spacing of these components is critical for the emission of gold particles from the gun’s nozzle. The third, perpendicular port of the T-connector connects the nozzle to the vacuum system described above. The small aperture of this nozzle, together with the use of a mounted laser guide (#6041100CD; Home Depot, Atlanta, GA) that identifies the center of the 1 mm^2^ target area on which gold particles will fall, permits microtargeting of specific target areas within retinal explants.

### Biolistic transfection with the Helios gun

Where noted, we used a conventional entrainment biolistic device (Helios Gene Gun System; Bio-Rad, Hercules, CA) to provide a comparison with transfections done using the modified capillary gun described above. The Helios device was used with a delivery pressure of 95 psi, with explants situated in 12-well plates positioned approximately 2.5 cm below the nozzle of the gun as previously described for brain slice explants [23,24].

### Microcarrier preparation

The biolistic devices described in this paper were used with standard 1.6 µm diameter gold microcarriers (Strem Chemicals, Inc., Kehl, Germany). We found that smaller particles (1 μm), which have been reported to preserve tissue integrity in the hour following transfection [25], do not readily penetrate into the RGC layer of explanted retinas using either the new or the Helios entrainment biolistic device. Gold particles were coated with an expression construct encoding yellow fluorescent protein (YFP) driven by the gWiz promoter, as previously described, using calcium/ethanol precipitation [24]. The particles were prepared identically for use in either the modified capillary gun or the entrainment device. For the modified capillary gun, particles were suspended in a fresh ethanol suspension at a concentration of 5 mg/ml. Particles prepared in this manner were stored at 4 °C for up to 2 weeks before use. For the Helios entrainment device, particles were loaded into Tefzel tubing and dried using a vacuum chamber before use.

### Explant preparation

These studies employed an ex vivo explant culture system that used intact retinal tissue from adult rats. Briefly, eyes were enucleated from adult CD Sprague-Dawley rats (Charles River, Wilmington, MA) immediately following application of profound anesthesia and euthanasia in accordance with NIH guidelines and under Duke IACUC approval and oversight. A circumferential cut was made 1 mm posterior to the limbus, then the retina was gently coaxed away from the posterior sclera to permit separation of the entire retina and optic nerve head from the scleral tissue via a single cut. Retinas were cut into sixths and placed RGC-side up onto an interface culture platform composed of filter paper (#2300 916; Sigma-Aldrich Co. LLC, St. Louis, MO) suspended in culture medium (Neurobasal medium supplemented with 0.5% Glutamax, 1% sodium pyruvate, 1% HEPES buffer, and 0.2% Primocin; Invitrogen, Carlsbad, CA). In these interface cultures, only the bottom surface of the explant was in direct contact with the culture medium, allowing efficient gas exchange via the upper surface of the tissue [26]. Explants were transfected using biolistics 30–60 min after dissection, after which time cultures were maintained in humidified incubators under 5% CO_2_ at 37 °C. Half of the medium was changed on the first day after dissection and on every second day thereafter.

### Assays of retinal ganglion cell morphology and function

RGC axonal morphology was assessed visually using a scoring system keying on morphological aspects of axonal pathology, notably the appearance, numbers, and sizes of axonal varicosities (“blebs”), and the overt loss of axonal continuity. An axonal bleb was defined as any axonal region that was more than twice as thick as the surrounding axon segment. Data are presented as the proportion of RGC axons in a given explant that did not exhibit axonal pathology.

RGC axonal transport capacity was assessed using Alexa 488- or 594-conjugated cholera toxin B (CTB; Invitrogen), a well established anterograde and retrograde neuronal tracer devoid of toxicity due to removal of the toxic α subunit [27,28]. To measure retrograde transport, explants were incubated overnight with a 0.05 μl drop of CTB (0.5 mg/ml) positioned on the surface of the retinal explant at the optic nerve head. As only the most distal portions of the explanted axons contacted the CTB, the number of peripheral RGC somata accumulating CTB signal via retrograde axonal transport served as a metric for axonal transport capacity. CTB-positive RGCs were quantified from a 25× image taken from the periphery of each explant using Matlab software (Mathworks, Inc., Natick, MA). Built-in Matlab filtering, segmentation, and thresholding functions were used to identify individual RGCs in an image, and the identified RGCs were then analyzed using the regionprops command.

Data are presented as means±standard error of the mean (SEM). Results from different groups were compared via a Student *t* test or ANOVA (ANOVA) followed by Dunn’s multiple comparison test. P values less than 0.05 were considered statistically significant. All experiments were repeated in at least three independent trials to ensure the reproducibility of any observed effects. All statistical comparisons were done using R 2.6.2 software (R Foundation for Statistical Computing, Vienna, Austria).

### Immunofluorescence assays

Immunohistochemistry was used to probe additional aspects of explant health. At the conclusion of experiments, explants were rinsed once in Dulbecco’s PBS (#D8537; Sigma-Aldrich Co. LLC) and fixed with 4% paraformaldehyde in PBS containing 4% sucrose for 1 h at room temperature. Retinas were then rinsed in PBS and blocked for 1 h in blocking solution (PBS containing 0.5% Triton-X 100, 1% BSA, and 10% normal goat serum). Tissues were incubated overnight in primary antibody solution (PBS containing 0.3% Triton-X 100, 0.5% BSA, and 10% normal goat serum) containing primary antibodies against green fluorescent protein (GFP; 1:1,500; Abcam, Cambridge, MA), glial fibrillary acidic protein (GFAP; 1:1,000; Millipore, Billerica, MA), or Smi-31 (1:1,000; Covance, Princeton, NJ), as indicated. Retinas were then rinsed three times (30 min each) in rinsing solution (PBS containing 0.1% Triton-X 100) and incubated for 1 h with secondary antibodies. Species-specific secondary IgG antibodies were conjugated to Alexa 488 or 594 fluorophores (Invitrogen) and diluted 1:1,000 in rinsing solution. Finally, retinas were washed three times (30 min each) in rinsing solution, mounted on microscope slides (Fisher Scientific, Pittsburgh, PA), coverslipped in Hydromount mounting medium (National Diagnostics, Atlanta, GA), and imaged as described below.

### Imaging

Photomicrographs were collected on a Zeiss fluorescence microscope (Carl Zeiss MicroImaging, LLC, Thornwood, NY) equipped with a charge-coupled device (CCD) camera (AxioCam MRm, Zeiss). Images of retinal explants were taken using Zeiss 5X Fluar (0.25 NA), Zeiss 10X Plan-Neofluar (0.3 NA), or Zeiss 25X Plan-Neofluar (0.8 NA oil) objectives, as noted. Postprocessing and quantification of images were performed using ImageJ (National Institutes of Health) and Matlab algorithms, respectively.

## Results

### Retinal ganglion cells in explanted retinas are labeled to their distal tips using the modified capillary gun

**Figure 2:**
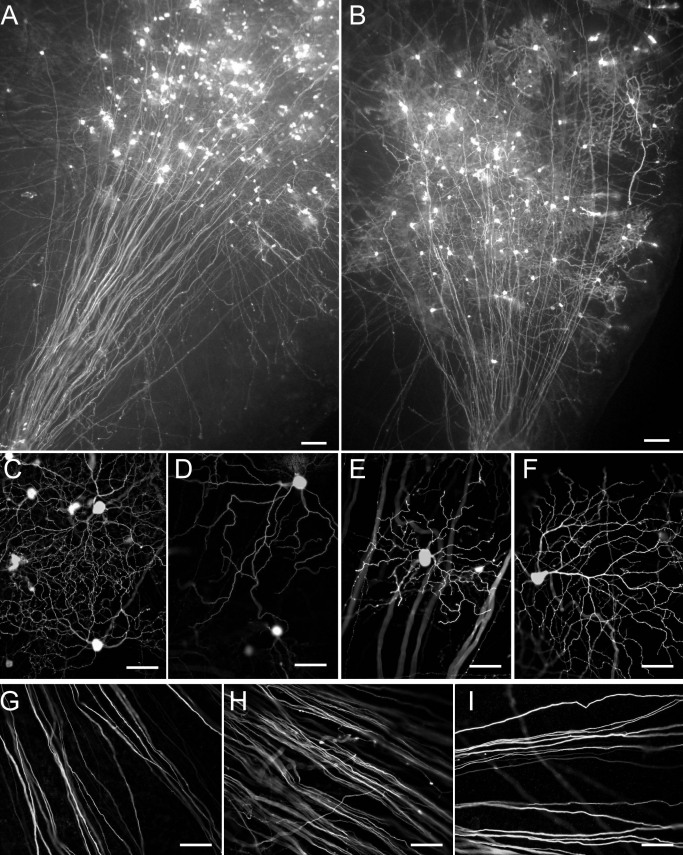
Transfection with the modified capillary gun labels retinal ganglion cells (RGCs) to their distal tips. **A**, **B**: Transfection of a retinal explant with YFP permits visualization of numerous RGCs and their axons, which course toward the optic nerve head. **C**-**F**: By 48 h after transfection, RGC somata and dendrites are fully labeled with fluorescent protein and show no evidence of blebs or degeneration after transfection with the modified capillary gun. **G**-**I**: The micro-targeting capacity of the gun permits clear visualization of YFP-filled RGC axons, which are smooth, straight, and devoid of varicosities in the region near the optic nerve head. Scale bar represents 100 μm in **A** and **B** and 50 μm in **C**-**I**.

Particle-mediated gene transfer was used to transfect RGCs in cultured explants with YFP to label them with a diffusible fluorescent marker. The modified capillary gun described here optimally transfects RGCs in retinal explants from adult rats (Figure 2A-B). RGCs may be disambiguated from displaced amacrine cells lying within the same layer by their extension of an axon traversing the nerve fiber layer to the optic nerve head. Transfected, YFP-expressing RGCs in these explants extended full dendritic (Figure 2C-F) and axonal (Figure 2G-I) arbors, which were filled with transfected fluorescent proteins to their distal tips, by 48 h after transfection. The number of cells transfected was proportional to the concentration of gold in the gold-ethanol suspension injected into the gun, with a gold concentration of 5 mg/ml sufficient to transfect RGCs reliably.

The modified microtargeting gun described here permits the transfection of a small area of the retina using the small aperture size of the gun’s nozzle. Cells transfected in a single “shot” typically span an area of approximately 1 mm^2^ in the retinal explant. This micro-targeting feature allows clear visualization of centripetal axons belonging to peripherally transfected RGCs, thereby facilitating quantification of axonal pathology. It also permits transfection of multiple, spatially segregated cell populations within a single explant with different constructs.

### Transfected retinal ganglion cells remain morphologically and functionally intact after transfection with the modified capillary gun

The health of RGCs is a critical issue in explant-based studies. To demonstrate that biolistic transfection using the modified capillary gun does not adversely affect the general health and viability of cells in the explanted retina, we assessed the morphology and function of RGCs in transfected explants over the course of 1 week ex vivo using a variety of measures, as described below.

**Figure 3:**
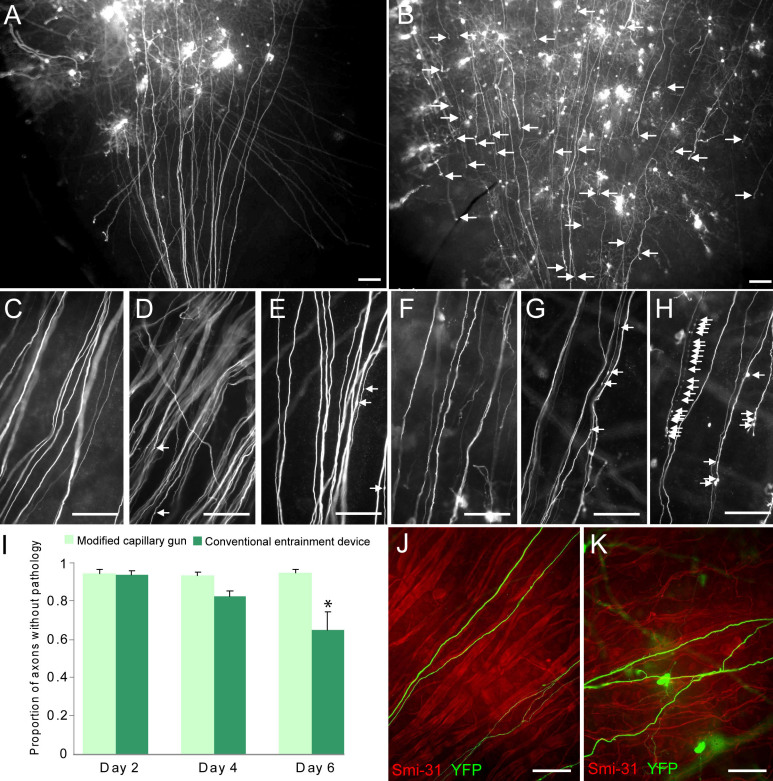
RGCs transfected with YFP using the modified capillary gun remain morphologically intact for 1 week ex vivo. **A**: Transfection with the modified capillary gun preserves RGC axons that extend from somata to the optic nerve head. **B**: In contrast, transfection with a conventional entrainment biolistic device produces many axons that lose continuity mid-explant (arrows). **C**-**E**: RGC axons remain healthy 2 (**C**), 4 (**D**), and 6 (**E**) days after transfection with the modified capillary gun. F-H: In contrast, those from explants transfected with the entrainment device are somewhat healthy on day 2 (**F**), but show overt degeneration by 4 (**G**) to 6 (**H**) days after transfection. **I**: Quantification of these results reveals that transfection with the entrainment device causes significantly greater degeneration than does transfection with the modified capillary gun. *p<0.05 by ANOVA. **J**: Smi-31 staining of explants transfected with the modified capillary gun reveals organized, bundled axons. **K**: In contrast, those in explants transfected with a conventional entrainment device show a disorganized axonal pattern. Scale bar represents 100 μm in **A** and **B** and 50 μm in **C**-**K**.

First, we found that RGCs in explanted retinas transfected with YFP using the modified capillary gun remained morphologically intact for up to one week after transfection (Figure 3). RGC axons extended unbroken from their origins at RGC somata to their tips at the optic nerve head (Figure 3A). For 2–6 days after transfection, these axons appeared smooth and straight, devoid of axonal varicosities and signs of Wallerian degeneration (Figure 3C-E). Additionally, immunostaining against Smi-31, which labels all RGC axons, demonstrated that RGC axons from explants transfected with the modified capillary gun remained healthy at the population level as well. Even 6 days after explant transfection, Smi-31 labeling showed RGC axons tightly organized into bundles coursing toward the optic nerve head (Figure 3J).

In contrast, RGC axons from explants transfected using a conventional entrainment biolistic device showed clear evidence of axonal stress and/or degeneration after transfection. Many RGCs transfected using this entrainment device had axons that terminated prematurely in the middle of the explant instead of extending fully to the optic nerve head (Figure 3B, arrows). As RGC axons lie close to the surface of the cultured explant, we suspect that the force of the helium jet from the conventional entrainment gun is sufficient to break the continuity of these axons. Additionally, although axons were initially somewhat healthy 2 days after transfection with the conventional entrainment device (Figure 3F), development of axonal varicosities was evident by 4 days (Figure 3G) and significantly worsened into overt degeneration by 6 days after transfection (Figure 3H; quantified in Figure 3I). Further, Smi-31 immunostaining 6 days after transfection revealed that axons were disorganized at the population level, having lost the bundled structure characteristic of the healthy retina by this time (Figure 3K).

To confirm these observations, we next examined whether morphologically intact RGCs were also functional after transfection with the modified capillary gun by probing their capacity for axonal transport. CTB is a widely used neuronal tracer that is actively taken up and transported in a retrograde manner. Only those RGCs that retain the ability to actively take up and transport the CTB tracer applied to their distal axons in the central retina will accumulate CTB in their somata. We could thus use CTB transport to RGC somata at the peripheral edge of retinal explants as a good metric of aggregate RGC functional capacity.

**Figure 4:**
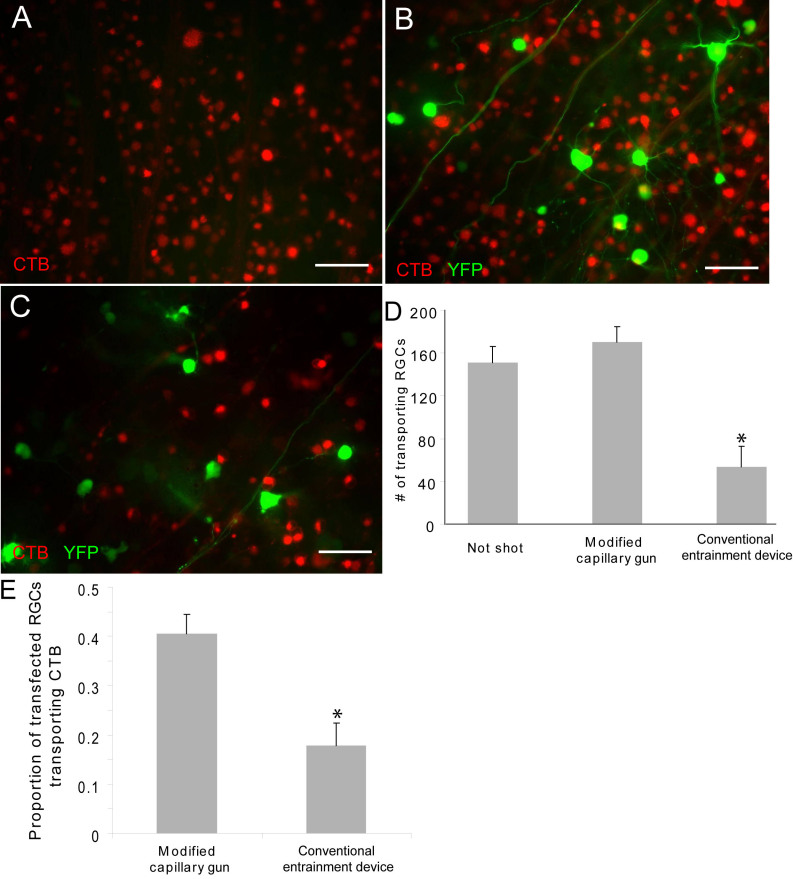
RGCs transfected with YFP using the modified capillary gun retain functional axonal transport. **A**, **B**: The number of RGCs functionally transporting a fluorescently labeled cholera toxic B (CTB) tracer and accumulating CTB in their somata does not differ between untransfected (**A**) and modified capillary gun-transfected (**B**) explants. **C**: In contrast, RGCs from explants transfected with a conventional entrainment biolistic device show marked disruption of axonal transport in comparison to non-transfected controls. **D**: Quantification of the number of cells transporting the tracer confirms significant impairment of transport capacity by the conventional entrainment device but not by the modified capillary gun. **E**: Quantification of the capacity of YFP-expressing RGCs to transport CTB also reveals significant impairment after transfection with the conventional entrainment biolostic device compared to the modified capillary gun. *p<0.05 by ANOVA. Scale bar represents 50 μm.

We assayed retrograde CTB transport 3 days after transfection with the modified capillary gun and a conventional entrainment biolistic device. As shown in Figure 4, retinal explants transfected using the modified capillary gun showed no significant impairment of CTB transport compared to nontransfected control explants (compare Figure 4A and Figure 4B; quantified in Figure 4D). In contrast, retrograde axonal transport was markedly reduced in explants transfected with the conventional entrainment device compared to nontransfected controls (compare Figure 4A and Figure 4C; quantified in Figure 4D). This impairment of axonal transport after transfection with the conventional entrainment device was notable for both the entire explanted RGC population (Figure 4A-D) as well as for YFP-labeled, transfected RGCs alone (Figure 4E). Additionally, we noted that transport in explants transfected with the conventional entrainment biolistic device tended to be patchy and inconsistent, with the worst transport generally being observed near the center of the explant where the helium blast was most severe.

### The glial microenvironment remains intact after retinal ganglion cell transfection using the modified capillary gun

**Figure 5:**
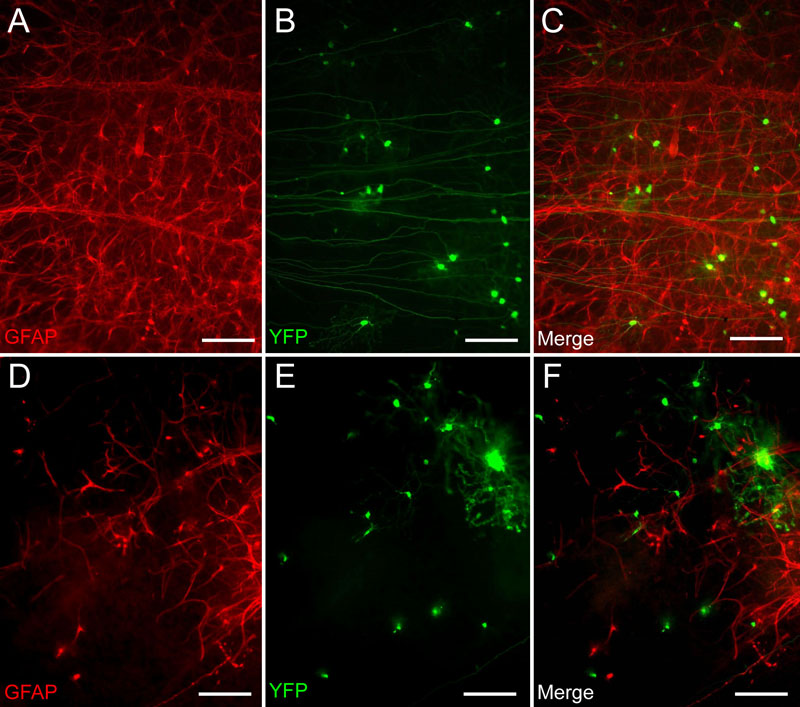
Glial microenvironment remains intact after RGC transfection using the modified capillary gun. **A**-**C**: The astroglial layers of retinal explants transfected with YFP using the modified capillary gun retain their normal, coherent, web-like appearance. **D**-**F**: In contrast, those in explants transfected using a conventional entrainment biolistic device show extended regions of astroglial degeneration. Scale bar represents 50 μm.

Finally, we used immunocytochemistry to examine the morphology of the astroglial cells that form the surface tissue layer in retinal explants. GFAP is a well known glial structural protein that becomes upregulated under conditions of glial activation. The astroglial web overlying the RGC layer showed no evidence of glial cell degeneration after transfection with the modified capillary gun. Indeed, the astrocytes making up this layer maintained a coherent, web-like structure in retinal explants even 6 days after transfection with the modified capillary gun (Figure 5A).

In contrast, the astroglial layer of retinal explants transfected with the conventional entrainment biolistic device frequently showed large areas marked by glial degeneration and loss after transfection (Figure 5D). This degeneration, which was observed in about half of the explants transfected, was typically accompanied by pathology in the underlying transfected retinal cells. As above, the most severe glial degeneration generally occurred near the center of the helium blast.

## Discussion

Here, we have presented a new device to deliver DNA-coated microparticles into the adult mammalian retina ex vivo with minimal damage to the tissue’s surface layers. By using low helium inflow pressures and vacuum diversion to eliminate helium emission in the transfection process, the current method allows efficient transfection as well as morphological and functional preservation of RGCs and their local microenvironment in adult retinal explants.

This method offers several advantages over existing methods. First, the modified capillary gun induces significantly less trauma in retinal explants than do conventional entrainment biolistic devices. As the RGC and overlying layer of astroglial cells are the most superficial layers of the retina, protection from transfection-induced trauma remains a critically important goal. As demonstrated by both morphological and functional preservation of RGC axons for at least 1 week ex vivo after transfection, reducing the trauma associated with gold microparticle delivery improves the health of transfected explants. Further, the new device also avoids damage to the astrocytic web overlying the RGC layer that would otherwise be caused by the helium impact of conventional entrainment devices. Given the increasingly appreciated role of glial interactions in neuronal function, preservation of the superficial glial layer of the retina is likely critical for maintaining the normal function and intercellular interactions of the retina.

Second, the small aperture size of the capillary gun permits microtargeting of specific regions of the retina as small as 1 mm^2^ in area, as was a feature of the original capillary gun design [21]. This targeting capacity enables transfection of single or multiple spatially segregated cell populations within a single explant, allowing several experimental conditions, including internal controls, to be included within a single retinal explant.

Finally, the modified capillary gun described here improves the speed and reproducibility of the transfection process. Because gold particles are suspended in ethanol instead of being dried onto tubing, particle preparation and shooting requires significantly less time than in the low-pressure transfection devices described in previous reports [21]. Additionally, the use of a gold suspension ensures that the gun emits a consistent and reproducible amount of gold in each shot. The particle injection system described here contains enough gold suspension for 50 shots before reloading is necessary, supporting more rapid and extended shooting sequences compared to tubing-based devices. Further, the use of a gold suspension eliminates variables such as drying times/conditions and inconsistent lot-to-lot Tefzel tubing surface properties, which significantly influence transfection efficiencies in conventional gene gun systems. Finally, the device described in detail here is easy and inexpensive to construct and requires no special machining techniques, making it a feasible alternative to the low-pressure capillary gun [21].

As with all biolistic transfection methods, the new device described here does not intrinsically support cell-type transfection specificity. However, this issue is less problematic in the explanted retina than in other tissue types. Because of the tightly structured layers of the retina, transfection can be optimized for a given layer (e.g., the RGC layer) by adjusting the height of the gun and helium inflow pressure. Within a narrow transfected layer, promoter-driven or morphology-based screening can then ensure that only the cell type(s) of interest are considered during analysis.

In summary, we have described a novel biolistic device that enables tissue microtargeting while preserving the microarchitecture of the retina for extended periods ex vivo. This device should be useful not only in the retina but also in other tissue explant settings in which preservation of local cellular and tissue integrity is a priority.

## References

[1] Rodieck RW, Brening RK (1983). Retinal ganglion cells: properties, types, genera, pathways and trans-species comparisons.. Brain Behav Evol.

[2] Sterling P (1983). Microcircuitry of the cat retina.. Annu Rev Neurosci.

[3] Johnson TV, Martin KR (2008). Development and characterization of an adult retinal explant organotypic tissue culture system as an in vitro intraocular stem cell transplantation model.. Invest Ophthalmol Vis Sci.

[4] Carter DA, Dick AD (2003). Lipopolysaccharide/interferon-gamma and not transforming growth factor beta inhibits retinal microglial migration from retinal explant.. Br J Ophthalmol.

[5] Satow T, Bae SK, Inoue T, Inoue C, Miyoshi G, Tomita K, Bessho Y, Hashimoto N, Kageyama R (2001). The basic helix-loop-helix gene hesr2 promotes gliogenesis in mouse retina.. J Neurosci.

[6] Sahaboglu A, Tanimoto N, Kaur J, Sancho-Pelluz J, Huber G, Fahl E, Arango-Gonzalez B, Zrenner E, Ekström P, Löwenheim H, Seeliger M, Paquet-Durand F (2010). PARP1 Gene Knock-Out Increases Resistance to Retinal Degeneration without Affecting Retinal Function.. PLoS ONE.

[7] Patzke C, Max KE, Behlke J, Schreiber J, Schmidt H, Dorner AA, Kröger S, Henning M, Otto A, Heinemann U, Rathjen FG (2010). The coxsackievirus-adenovirus receptor reveals complex homophilic and heterophilic interactions on neural cells.. J Neurosci.

[8] Escande-Beillard N, Washburn L, Zekzer D, Wu ZP, Eitan S, Ivkovic S, Lu Y, Dang H, Middleton B, Bilousova TV, Yoshimura Y, Evans CJ, Joyce S, Tian J, Kaufman DL (2010). Neurons preferentially respond to self-MHC class I allele products regardless of peptide presented.. J Immunol.

[9] Escande-Beillard N, Washburn L, Zekzer D, Wu ZP, Eitan S, Ivkovic S, Lu Y, Dang H, Middleton B, Bilousova TV, Yoshimura Y, Evans CJ, Joyce S, Tian J, Kaufman DL (2010). Neurons preferentially respond to self-MHC class I allele products regardless of peptide presented.. J Immunol.

[10] Patzke C, Max KE, Behlke J, Schreiber J, Schmidt H, Dorner AA, Kröger S, Henning M, Otto A, Heinemann U, Rathjen FG (2010). The coxsackievirus-adenovirus receptor reveals complex homophilic and heterophilic interactions on neural cells.. J Neurosci.

[11] Sahaboglu A, Tanimoto N, Kaur J, Sancho-Pelluz J, Huber G, Fahl E, Arango-Gonzalez B, Zrenner E, Ekström P, Löwenheim H, Seeliger M, Paquet-Durand F (2010). PARP1 Gene Knock-Out Increases Resistance to Retinal Degeneration without Affecting Retinal Function.. PLoS ONE.

[12] Lo DC, McAllister AK, Katz LC (1994). Neuronal transfection in brain slices using particle-mediated gene transfer.. Neuron.

[13] Arnold D, Feng L, Kim J, Heintz N (1994). A strategy for the analysis of gene expression during neural development.. Proc Natl Acad Sci USA.

[14] Jiao S, Cheng L, Wolff JA, Yang NS (1993). Particle bombardment-mediated gene transfer and expression in rat brain tissues.. Biotechnology (N Y).

[15] Rockhill RL, Daly FJ, MacNeil MA, Brown SP, Masland RH (2002). The diversity of ganglion cells in a mammalian retina.. J Neurosci.

[16] Kettunen P, Demas J, Lohmann C, Kasthuri N, Gong Y, Wong RO, Gan WB (2002). Imaging calcium dynamics in the nervous system by means of ballistic delivery of indicators.. J Neurosci Methods.

[17] Moritoh S, Tanaka KF, Jouhou H, Ikenaka K, Koizumi A (2010). Organotypic tissue culture of adult rodent retina followed by particle-mediated acute gene transfer in vitro.. PLoS ONE.

[18] Sanford JC, Klein TM, Wolf ED, Allen N (1987). Delivery of substances into cells and tissues using a particle bombardment process.. Particulate Science and Technology: An International Journal.

[19] Donovan SL, Dyer MA (2006). Preparation and square wave electroporation of retinal explant cultures.. Nat Protoc.

[20] Matsuda T, Cepko CL (2004). Electroporation and RNA interference in the rodent retina in vivo and in vitro.. Proc Natl Acad Sci USA.

[21] Rinberg D, Simonnet C, Groisman A (2005). Pneumatic capillary gun for ballistic delivery of microparticles.. Appl Phys Lett.

[22] Washbourne P, McAllister AK (2002). Techniques for gene transfer into neurons.. Curr Opin Neurobiol.

[23] Yacoubian TA, Lo DC (2000). Truncated and full-length TrkB receptors regulate distinct modes of dendritic growth.. Nat Neurosci.

[24] Lo DC (2001). Neuronal transfection using particle-mediated gene transfer.. Curr Protoc Neurosci.

[25] Roizenblatt R, Weiland JD, Carcieri S, Qiu G, Behrend M, Humayun MS, Chow RH (2006). Nanobiolistic delivery of indicators to the living mouse retina.. J Neurosci Methods.

[26] Stoppini L, Buchs PA, Muller D (1991). A simple method for organotypic cultures of nervous tissue.. J Neurosci Methods.

[27] Angelucci A, Clasca F, Sur M (1996). Anterograde axonal tracing with the subunit B of cholera toxin: a highly sensitive immunohistochemical protocol for revealing fine axonal morphology in adult and neonatal brains.. J Neurosci Methods.

[28] Luppi PH, Fort P, Jouvet M (1990). Iontophoretic application of unconjugated cholera toxin B subunit (CTb) combined with immunohistochemistry of neurochemical substances: a method for transmitter identification of retrogradely labeled neurons.. Brain Res.

